# Evidence for Model-based Computations in the Human Amygdala during Pavlovian Conditioning

**DOI:** 10.1371/journal.pcbi.1002918

**Published:** 2013-02-21

**Authors:** Charlotte Prévost, Daniel McNamee, Ryan K. Jessup, Peter Bossaerts, John P. O'Doherty

**Affiliations:** 1 Trinity College Institute of Neuroscience and School of Psychology, Dublin, Ireland; 2 Division of Humanities and Social Sciences, California Institute of Technology, Pasadena, California, United States of America; 3 Computation and Neural Systems Program, California Institute of Technology, Pasadena, California, United States of America; 4 Department of Management Sciences, Abilene Christian University, Abilene, Texas, United States of America; Indiana University, United States of America

## Abstract

Contemporary computational accounts of instrumental conditioning have emphasized a role for a model-based system in which values are computed with reference to a rich model of the structure of the world, and a model-free system in which values are updated without encoding such structure. Much less studied is the possibility of a similar distinction operating at the level of Pavlovian conditioning. In the present study, we scanned human participants while they participated in a Pavlovian conditioning task with a simple structure while measuring activity in the human amygdala using a high-resolution fMRI protocol. After fitting a model-based algorithm and a variety of model-free algorithms to the fMRI data, we found evidence for the superiority of a model-based algorithm in accounting for activity in the amygdala compared to the model-free counterparts. These findings support an important role for model-based algorithms in describing the processes underpinning Pavlovian conditioning, as well as providing evidence of a role for the human amygdala in model-based inference.

## Author Summary

A hot topic in the neurobiology of learning is the idea that there may be two distinct mechanisms for learning in the brain: a model-based learning system in which predictions are made with respect to a rich internal model of the learning environment, versus a “model-free” mechanism in which trial-and-error learning occurs without any rich internal representation of the world. While the focus in the literature to date has been on the role of these mechanisms in instrumental conditioning, almost nothing is known about whether more fundamental kinds of learning such as Pavlovian conditioning also involve model-based processes. Furthermore, nothing is known about the extent to which the amygdala, which is known to be a core structure for Pavlovian learning, contains neural signals consistent with a model-based mechanism. To address this question, we used a novel Pavlovian conditioning task and scanned human volunteers with a special high-resolution fMRI sequence that enabled us to obtain signals within the amygdala with over four times the resolution of conventional imaging protocols. Using this approach in combination with sophisticated computational analyses, we find evidence to suggest that the human amygdala is involved in model-based computations during Pavlovian conditioning.

## Introduction

Neural computations mediating instrumental conditioning are suggested to depend on two distinct mechanisms: a model-based reinforcement learning system, in which the value of actions are computed on the basis of a rich knowledge of the states of the world and the nature of the transitions between states, and a “model-free” reinforcement learning system in which action-values are updated incrementally via a reward prediction error without using a rich representation of the structure of the decision problem [1]–[6]. Accumulating evidence supports the existence of model-based representations during instrumental conditioning in a number of brain regions, including the ventromedial prefrontal cortex, striatum and parietal cortex [7]–[9]. However, instrumental conditioning is not the only associative learning mechanism in which model-based computations might play a role.

Pavlovian conditioning can also be framed as a model-based learning process, in which the animal begins with a model of the possible structure of the world: the stimuli within it, and sets of possible contingencies that could exist between conditioned stimuli and unconditioned stimuli, as well as assumptions about how these contingencies might change over time. In essence, learning within such a system corresponds to determining the statistical evidence for which structure out of the set of possible causal structures best describes the environment, as well as determining whether or when the relevant causal processes have changed as a function of time. Model-based approaches to classical conditioning to date have used Bayesian methods to yield inference over structure space [10].

**Figure 1:**
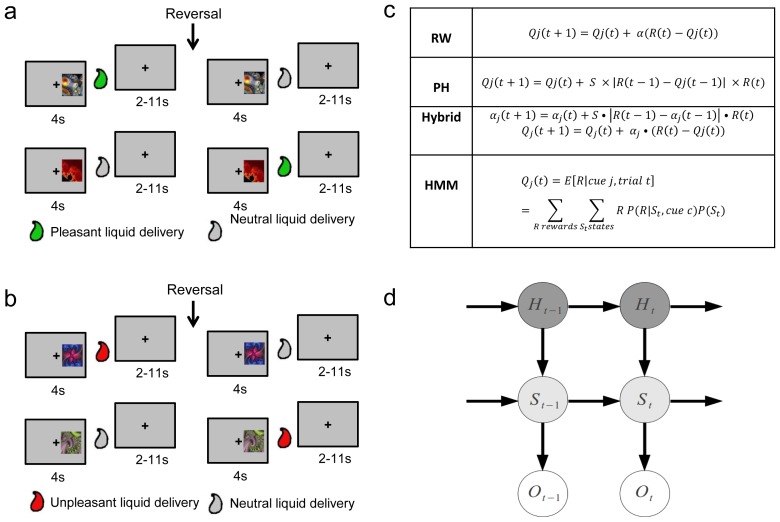
Task and equations. **a**, Appetitive Pavlovian learning task. **b**, Aversive Pavlovian learning task. Sequence and timing of events in the appetitive and aversive sessions are shown. On each trial, a cue was presented on one side of the screen for 4 s, followed by some liquid delivery 60% of the time. The trial ended with a 2–11 s inter-trial interval. Each session started with the presentation of cue 1 and cue 2, leading 60% of the time to a pleasant or a neutral liquid delivery in the appetitive session or an unpleasant or a neutral liquid delivery in the aversive session. After a number of trials, a reversal occurred so that cue 1 now led to the liquid associated with cue 2, and cue 2 led to the liquid associated with cue 1. Subsequently, a new pair of cues was presented, which also reversed after a number of trials. In total, three new pair of cues were presented, and each of these pairs reversed once. **c**, Computational models used to estimate expected reward on each trial (Qj). The expected rewards generated by the model-free learning algorithms (Rescorla-Wagner (RW), Pearce-Hall (PH) and the Hybrid models) were compared against a model-based learning algorithm (Hidden Markov Model or HMM) at both the behavioral and neural levels. **d**, Graphical model representation of the Bayesian HMM.

Very little is known about the extent to which such model-based algorithms are implemented in the brain during Pavlovian conditioning. The aim of the present study was to address this question using computational fMRI. Human participants were scanned while undergoing a Pavlovian conditioning procedure with a sufficiently complex structure to enable the predictions of model-based and model-free algorithms to be compared and contrasted (see Figure 1). We then constructed a Bayesian algorithm incorporating a model of the structure of the learning problem and compared the predictions of this algorithm against two widely adopted prediction-error driven “model-free” algorithms for Pavlovian conditioning: the Rescorla-Wagner (RW) learning rule [11] and the Pearce-Hall (PH) learning rule [12] as well as a recently developed model which combines the two: the Hybrid model [13].

In order to test for model-based signals in the brain we focused on the amygdala, a structure heavily implicated in Pavlovian conditioning in both animal and human studies [14]–[17]. To obtain signals from this region with sufficient fidelity, we used a high-resolution fMRI protocol in which we acquired images with more than four times the resolution of a standard 3 mm isotropic scan, alongside an amygdala specific normalization procedure [18]. We hypothesized that the model-based algorithm would account better for both behavioral and fMRI data acquired during both the appetitive and aversive conditioning phases than would the models of Pavlovian conditioning which do not contain such structured knowledge.

## Results

### Behavioral results

#### Affective ratings for the liquid outcomes

**Figure 2:**
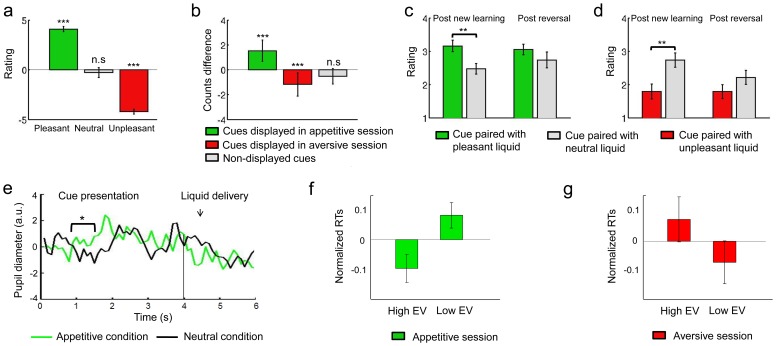
Behavioral results. **a**, Ratings for the pleasant, neutral and unpleasant liquids (−5 being very unpleasant and 5 very pleasant). *** indicates a significance of p<0.001 as computed by one sample t-tests comparing the mean of the different liquids against a mean of 0, n.s stands for not significant. **b**, Difference in the number of times a cue is preferred after - before the experiment. *** indicates a significance of p<0.001 as computed by one sample t-tests comparing the mean of the different liquids against a mean of 0. **c,d** Ratings for the cue paired with the pleasant or unpleasant liquid and the cue paired with the neutral liquid after a few trials after a new pair of cue has been presented (Post new learning) and a few trials after a reversal has occurred (Post reversal). A rating of 1 indicates that participants strongly dislike the cue whereas a rating of 4 indicates that they strongly like it. ** indicates a significance of p<0.01 as computed by two sample t-tests comparing the means of the ratings for the cues paired with pleasant/unpleasant and neutral liquids. **e–g**, Conditioned responses. **e**, Time course for pupil diameter in response to cues paired with the pleasant liquid (green line) and the neutral liquid (black line) averaged across all trials in the appetitive session for the 10 subjects showing reliable amplitude in their pupil diameter. A one-tailed paired t-test for a time window 0.8–1.5 s revealed a significant decrease in constriction when participants were presented with cues paired with the pleasant liquid (p<0.05). **f,g**, Normalized response times averaged across low and high categories of expected values (EV) as determined by the model-based learning algorithm in the appetitive and aversive sessions. Expected values are split into two bins at 0.3 for the appetitive session (with on average 57+/−4.8 trials in high EV bin, 62+/−4.8 trials in low EV bin) and −0.3 for the aversive session (with on average 48+/−3.5 trials in high EV bin, and 62+/−3.5 trials in low EV bin).

Subjects were asked to give subjective ratings of the pleasant and neutral tasting liquids before and after the appetitive session and of the unpleasant and neutral tasting liquids before and after the aversive session. The pleasant, neutral and unpleasant tasting liquids (unconditioned stimuli or USs) were reported to be highly pleasant, neutral and unpleasant by subjects as indicated by their ratings averaged across before and after conditioning (Figure 2a). There was no significant difference in the pleasantness ratings of any of the liquid outcomes before and after conditioning (paired t-tests, all p>0.05).

#### Revealed preference rankings for the cue stimuli

Subjects made binary preferences between the visual cues used in the conditioning protocols before and after the experiment (Figure 2b). Subjects showed increased preference rankings for the cues displayed in the appetitive session (averaging across both CS+ and CS− cues as both were paired with reward and neutral outcomes over the course of the experiment due to the reversal) after as compared to before the experiment (p<0.001). Furthermore, the set of cues used in the aversive sessions showed a significant decrease in their relative preference rankings (p<0.001). Preference rankings for the control cues (cues not included in either the appetitive or aversive conditioning sessions) showed no significant changes from before to after the experiment. These results indicate that while the cues displayed in the appetitive session have acquired an increased positive value, those displayed in the aversive session have acquired a negative value; indicating that subjects showed a modulation in their affective responses to the cue stimuli as a function of the context in which these stimuli had been conditioning (appetitive versus aversive).

#### Pleasantness ratings for the cue stimuli

We also obtained pleasantness ratings from subjects while in the scanner during the conditioning procedure. In the middle of the appetitive session, a few trials after a new pair of cue was presented, subjects rated the cue paired with the pleasant liquid significantly higher than the cue paired with the neutral liquid (p<0.01) (Figure 2c). Subjective ratings were obtained at the end of the appetitive session, hence following reversal of the last pair of cues and although they still rated the cue paired with the pleasant liquid higher than the one paired with the neutral liquid, this difference was not significant. Similarly, in the aversive session, the cue paired with the unpleasant liquid was rated significantly higher than the cue paired with the neutral liquid a few trials after a novel pair of cue was presented (p<0.01) but not after a reversal had occurred (Figure 2d).

#### Heart rate

Participants' pulse rate (an estimation of heart rate) was monitored using a pulse oximeter for the duration of the experiment. Existing research on heart rate responses to significant stimuli has identified an initial bradycardia associated with more aversive stimuli [19]. This deceleration is thought to express attentional orienting to salient events through parasympathetic activity [20]. Aversive trials were associated with a more pronounced cardiac deceleration (as assessed by the number of beats) compared to appetitive trials during anticipation, in a time window of 1.5–3.5 s following stimulus onset, as reported elsewhere [21] (paired t-test, p<0.01). Such physiological changes signal a more aversive emotional state for aversive as compared to appetitive trials, thereby reflecting a differential heart-rate conditioned response in the aversive relative to the appetitive conditioning trials.

#### Respiration

When analyzing respiration signals, we found that in the aversive condition, subjects learned to inspire before cue offset and expire at the time of the aversive liquid delivery. In contrast, subjects expired before cue offset and inspired at the time of the appetitive liquid delivery in the appetitive condition. The amplitudes between the appetitive and aversive conditions were significantly different both before cue offset (3.5 s) and at the time of liquid delivery (4.5 s) (p<0.05). However, note that these results need to be interpreted with caution because they do not survive multiple comparisons across all time windows tested.

#### Pupil dilation and blinking

We also recorded pupil diameter, an automatic measure of arousal previously shown to provide a measure of conditioning [22]–[24]. We found a significantly smaller amplitude in pupil diameter for trials where the cue was predictive of the pleasant liquid (appetitive condition) as compared to trials where the cue was predictive of the neutral liquid (neutral condition) (p<0.05) in a time window of 0.8–1.5 s after cue onset where amplitude changes in pupil diameter have previously been reported [23] in the 10 subjects from which we obtained pupil amplitude measures (Figure 2e). A higher degree of arousal (significantly smaller peak amplitude) would have been equally expected when subjects saw cues predictive of the aversive liquid; however, reliable analysis of amplitude in pupil diameter for these trials was prevented by the prolonged blinking elicited by these aversive cues. Given that blinking is also a conditioned response, we looked for evidence of blinking in the aversive condition as opposed to the neutral condition. We found significant differences between the aversive and neutral conditions during the first second after cue onset and last second before cue offset (paired t-tests, p<0.05) as well as at the time of liquid delivery and swallowing (paired t-tests, p<0.01).

#### Model comparison on behavioral data using reaction times

We used the Bayesian information criterion (BIC) approximation as the model evidences in order to compare the model goodness of the model-based HMM against the prediction error based model-free algorithms in a Bayesian Model Selection (BMS) analysis on the basis of trial by trial variation in reaction times (Table 1). For validation, we also compared these models to a baseline model (Table 2). We found that the model-based HMM fit better than each of the other algorithms that were tested including the baseline model, indicating that this algorithm was providing the best account of trial by trial variation in conditioning as reflected in reaction times. On the other hand, neither the RW nor the PH learning rules provided a better fit to the data than did the baseline model, suggesting that these algorithms cannot account for changes in reaction time as a function of conditioning any better than a random actor (Table 1 and 2). Note that we also constructed a simpler reduced-model version of our HMM which functioned in a manner more closely resembling a “model-free” algorithm. The essential difference between these two HMMs is that the reduced-model HMM does not incorporate knowledge of when contingencies are expected to reverse. The EV signals from these two models are essentially indistinguishable as they are almost completely correlated (r = 0.987 in the appetitive session, r = 0.986 in the aversive session). Hence, we did not include the reduced-model HMM EV signal in the behavioral model comparison described above, and therefore cannot rule out this type of algorithm based on the behavioral data alone. Instead we must turn to the neural data to discriminate these two possible accounts (see below).

**Table 1:** Behavioral BMS analysis.

	HMM	RW	PH	Hybrid
**Appetitive session**	xp = 0.99	xp = 3×10^−6^	xp = 4×10^−6^	xp = 6×10^−6^
	pp = 0.84	pp = 0.05	pp = 0.06	pp = 0.05
**Aversive session**	xp = 0.99	xp = 3×10^−3^	xp = 2.9×10^−5^	xp = 2.1×10^−5^
	pp = 0.71	pp = 0.19	pp = 0.05	pp = 0.05

Behavioral Bayesian Model Selection (BMS) analysis using the BIC approximation as the model evidences across model-based (HMM) and prediction-error driven model-free (RW, PH and Hybrid) learning algorithms. xp represent exceedance probabilities, pp represent posterior probabilities.

**Table 2:** Model validation.

	HMM<Baseline	RW<Baseline	PH<Baseline	Hybrid<Baseline
**Aversive Session**	<0.05	0.93	1	1
**Appetitive Session**	<0.001	1	1	1

A random effects test of the models versus a baseline model was performed by simulating random expected value estimates (10,000 repetitions) and then computing a non-parametric p-value per subject as the fraction of repetitions in which the baseline BIC is lower than the model BIC (indicating a better fit). These p-values were then combined across subjects using Fisher's combined probability test. Only HMM outperforms the baseline model in both the appetitive and aversive sessions.

The normalized RT data is shown plotted against the value signal predictions of the HMM model in Figure 2f,g, indicating that RTs become slower under situations where the cue presented is associated with a stronger prediction of an aversive outcome in the aversive condition, and become faster as cues are associated with a stronger prediction of an appetitive outcome in the appetitive condition.

### fMRI results

We report results from our analyses based on our model-based learning algorithm (the HMM model) within the amygdala using a height threshold of p<0.005, with an extent threshold significant at p<0.05 corrected for multiple comparisons. We first report signals correlating with signals generated by our model-based HMM, and then we compare the performance of our model-based algorithm against its model-free counterparts in terms of the capacity of these models to account for BOLD activity in the amygdala.

#### Expected value signals

**Figure 3:**
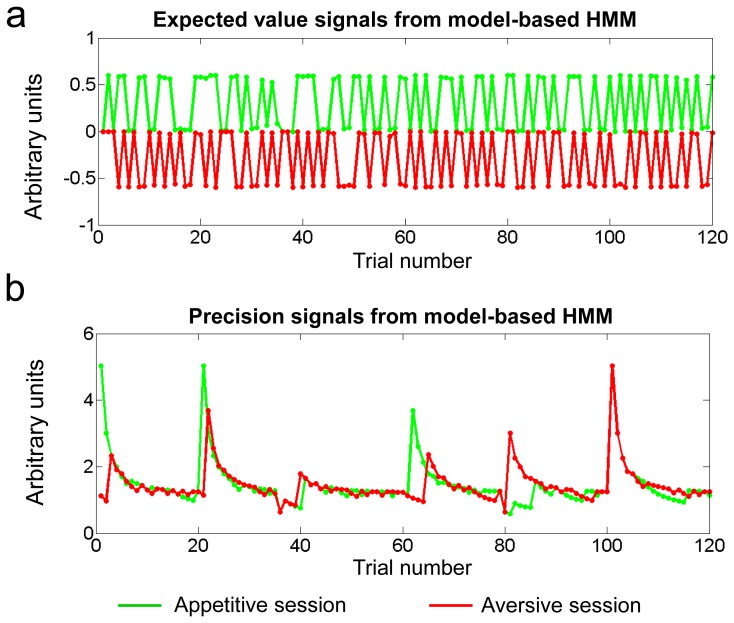
Expected value and precision signals. **a**, Plots showing expected value signals. **b**, Precision signals from the model-based learning algorithm for the appetitive (green) and aversive (red) sessions for a typical participant.

**Figure 4:**
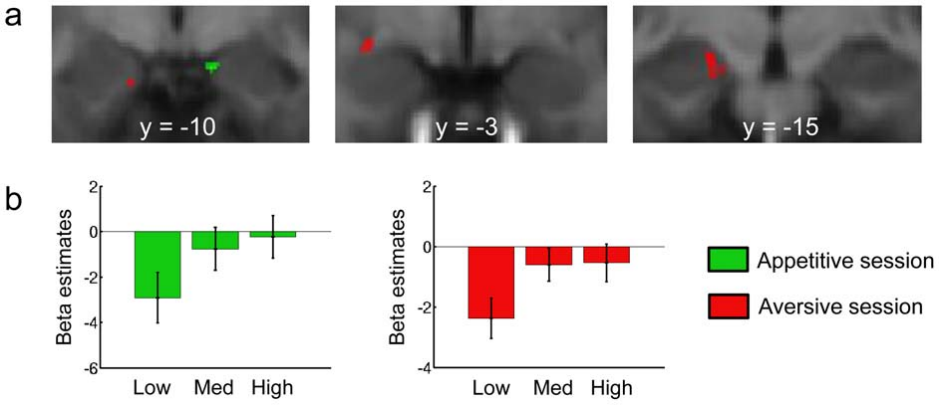
Expected value signals from the model-based learning algorithm model in the amygdala. **a**, Blood oxygen level-dependent (BOLD) signals positively correlating with the magnitude of the expected value of the cue were found in the basolateral complex in the appetitive session (in green) and in the centromedial complex in the aversive session (in red). **b**, Plots showing the beta estimates for low, medium and high categories of expected rewards in the appetitive (green) and aversive (red) sessions in the clusters activated using the leave-one out method.

We first investigated BOLD activity in the amygdala correlating with expected value (EV) signals at the time of cue presentation (see Figure 3 a for an illustration of EV signals). In the appetitive session, we found significant activity positively correlating with expected value in the medial part of the right amygdala, corresponding to the basolateral complex (Figure 4a in green, MNI [x y z] [10 −10 −18], T = 6.29, k = 28 voxels). In the aversive session, activity positively correlating with expected value was found in the centromedial complex of the left amygdala (Figure 4a in red, [x y z] [−27 −2 −9], T = 5.63, k = 44 voxels; [x y z] [−17 −15 −14], T = 5.41, k = 69 voxels), such that the greater the activity in these areas, the less an aversive outcome is predicted to occur. We also looked for areas correlating negatively with EV in both the appetitive and aversive sessions, that is, areas showing an increase in activity the less a positive outcome was predicted to occur given the cue. We did not find evidence for such activity in the amygdala in either the appetitive or the aversive session at our statistical threshold.

#### Precision signals

**Figure 5:**
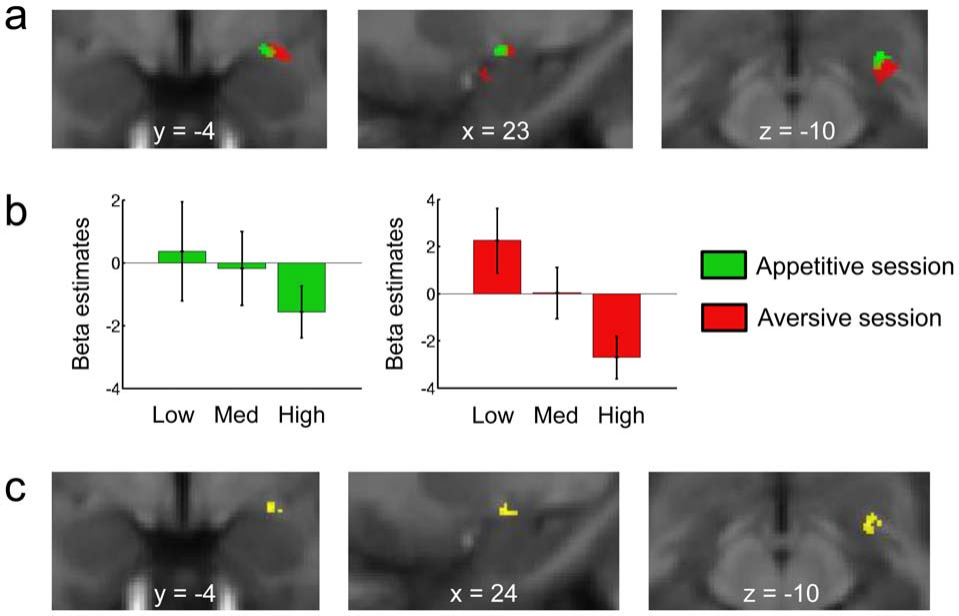
Precision signals from the model-based learning algorithm in the amygdala. **a**, Blood oxygen level-dependent (BOLD) signals correlating with the precision of the cue were found in the centromedial complex of the amygdala in both the appetitive session (in green) and aversive session (in red). **b**, Plots showing the beta estimates for low, medium and high categories of precision in the appetitive (green) and aversive (red) sessions in the clusters activated using the leave-one out method. **c**, Results from formal conjunction analysis of precision signals from the appetitive and aversive sessions in the centromedial complex.

Next, we examined amygdala activity correlating positively with precision or else correlating negatively with precision during both the appetitive and aversive sessions (see Figure 3b for an illustration of precision signals). While no significant negative correlation was found with precision, we did find significant positive correlations with precision signals during both the appetitive and aversive sessions within our centromedial complex ROI (appetitive session: [x y z] [25 −1 −10], T = 4.12, k = 44; aversive session: [x y z] [27 −5 −10], T = 5.31, k = 115; [x y z] [18 −2 −16], T = 4.75, k = 44)(Figure 5a). To test whether there was a significant overlap between these clusters in the appetitive and aversive sessions, we performed a formal conjunction analysis (at our omnibus threshold of p<0.005 with a cluster extent of p<0.05). In this contrast we found a common area activated by precision signals in the appetitive and aversive sessions in the centromedial complex of the amygdala ([x y z] [24 −4 −9], T = 3.52, k = 23) (Figure 5c).

#### Model-comparison on BOLD data

In order to determine whether BOLD activity in the amygdala is better accounted for by the model-based HMM than by the prediction-error driven model-free learning algorithms, we performed a Bayesian Model Selection (BMS) analysis (Table 3). The expected value contrasts from our model-based hidden state Markov switching model (HMM) and the prediction error driven “model-free” Rescorla-Wagner (RW), Pearce-Hall (PH) and Hybrid models were used to compare BOLD activity in the amygdala separately for the aversive and appetitive sessions. In this model comparison, we included voxels within a 4 mm sphere centered on the peak voxels of amygdala activities correlating with either expected value signals for the model-based HMM or expected value signals for the model-free algorithms using the leave-one out method, thereby avoiding a non-independence bias in the voxel selection. We found that the model-based HMM outperformed all prediction-error driven model-free algorithms with an exceedance probability of 0.94 (posterior probability = 0.64) for the aversive session and of 0.93 (posterior probability = 0.55) for the appetitive session.

**Table 3:** Neural BMS analysis.

	HMM	RW	PH	Hybrid
**Appetitive session**	xp = 0.93	xp = 0.05	xp = 7×10^−3^	xp = 0.02
	pp = 0.50	pp = 0.21	pp = 0.12	pp = 0.17
**Aversive session**	xp = 0.95	xp = 7.9×10^−5^	xp = 0.05	xp = 1.5×10^−4^
	pp = 0.61	pp = 0.05	pp = 0.28	pp = 0.06

Neural Bayesian Model Selection (BMS) analysis in the amygdala for Expected value signals generated by the model-based (HMM) and prediction-error driven model-free (RW, PH and Hybrid) learning algorithms. xp represent exceedance probabilities, pp represent posterior probabilities.

As noted earlier, we also constructed a “reduced-model” version of our HMM. While the EV signals generated by the model-based and reduced-model HMM are virtually identical, the precision signals are in fact quite distinct, enabling the predictions of these two models to be compared against the neural data. We extracted BOLD activity within the contrasts showing activity positively correlating with precision signals including voxels within a 4 mm sphere centered on the peak voxels of the amygdala activities correlating either with precision signals for the model-based HMM or the “model-free” HMM using the leave-one out method, thereby avoiding a non-independence bias in the voxel selection. We found that activity was better explained by precision signals estimated by the model-based HMM in both the aversive and appetitive sessions (aversive session: exceedance probability = 0.99, posterior probability = 0.75; appetitive session: exceedance probability = 0.68, posterior probability = 0.55). Thus, our fMRI findings in the amygdala clearly support the superiority of our model-based HMM over the reduced model alternative, especially in the aversive session.

## Discussion

In this study, we used a Pavlovian conditioning task with a rudimentary higher-order structure in both appetitive and aversive domains to investigate whether neural activity in the human amygdala reflects learning that requires access to model-based representations. By comparing neural activity correlating with expected value signals generated by model-based versus model-free learning algorithms using a Bayesian model selection (BMS) procedure, we have been able to show that in at least some parts of the human amygdala activity during Pavlovian conditioning is better accounted for by a model-based algorithm rather than by prediction error driven model-free algorithms.

One of the critical distinctions between the prediction error driven model-free and model-based learning algorithms in the present study is that while the expected value of a stimulus previously paired with the unpleasant outcome is still low following reversal of contingencies because that was the value it had before reversal in a model-free system, the expected value of this stimulus will become high in a model-based system because it incorporates the knowledge that after a reversal, stimulus values switch (i.e. there is full resolution of uncertainty when a reversal occurs). We have captured model-based representations in formal terms using an elementary Bayesian hidden Markov computational model that incorporates the task structure (by encoding the inverse relationship between the cues and featuring a known probability that the contingencies will reverse).

Our behavioral analysis demonstrated that participants showed evidence of conditioned responses to the conditioned stimuli and thus successfully learnt the associations between the different cues and outcomes. In a trial-by-trial analysis in which we correlated reaction times against the model predictions, we found that the HMM model predicted changes in reaction times over time as a function of learning better than the prediction-error driven model-free alternatives, and that the prediction error model-free algorithms did not predict variation in reaction times significantly better than chance.

In the neuroimaging data, we found trial-by-trial positive correlations of model-based expected values in an area consistent with the basolateral complex of the amygdala according to the Mai atlas in the appetitive session, and in areas in the likely vicinity of the centromedial complex in the aversive session [25]. It is interesting to note that activity in these same areas (i.e. basolateral versus centromedial complex) has been found to correlate with expected value signals generated by a simple RW model in a recent reward versus avoidance instrumental learning task (in an appetitive versus aversive context respectively) [18]. Using a BMS procedure, we found that amygdala activity correlating with expected value was best explained by the model-based than by the prediction error driven model-free learning algorithms. Whereas the model-free system has received considerable attention in the past [26], the more sophisticated and flexible model-based system, has been more sparsely studied particularly in relation to its role in Pavlovian learning. Thus, our results point to the need for integrating model-based representations and their rich adaptability into our understanding of Pavlovian conditioning in general, and of the role of the amygdala in implementing this learning process in particular.

Another important feature of the model-based algorithm featured in this study, is that as well as keeping track of expected value, this model also keeps track of the degree of precision in the prediction of expected value over the course of learning. This precision starts off low at the beginning of a learning session with a new stimulus because the expected value computation is very uncertain at this juncture, but once outcomes are experienced in response to specific cues, the precision in the estimate quickly increases. However, this precision lessens again as the trial progresses because a reversal in the contingencies is increasingly expected to occur (hence the expected value becomes more and more uncertain). Signals correlating with precision were found to be located in the vicinity of the centromedial complex in both the appetitive and aversive sessions. Precision signals might play an important role in the directing of attentional resources toward stimuli in the environment. The presence of a precision signal in the centromedial amygdala in the present paradigm could be a key computational signal underpinning the putative role of this structure in directing attention and orienting toward affectively significant stimuli.

The presence of a precision-related signal in the amygdala during Pavlovian conditioning may relate to other findings in which the amygdala has been suggested to play a role in “associability” as implemented in a model-free algorithm such as the Pearce-Hall learning rule [13], [27]. Associability as defined in such a model is essentially a model-free computation of uncertainty, the inverse of precision: associability is maximal when the absolute value difference between expected and actual rewards is greatest. However, in our case, an associability signal is clearly distinct from the signal we observe in the amygdala in the centromedial complex (even leaving aside the fact the signal we found is negatively as opposed to positively correlated with uncertainty). First of all, because the signal in our HMM is model-based, it changes to reflect anticipated changes in task structure (such as a reversal), whereas Pearce-Hall associability does not change to reflect anticipated changes in task structure, both rather changes only reflexively once contingencies have reversed.

Further evidence that the amygdala is involved in model-based computations came from an additional analysis in which we compared the signals generated by our model-based HMM against signals generated by a reduced version of our HMM in which knowledge of when contingencies are expected to reverse was not incorporated. Although this reduced model still generated very similar expected value signals as the model-based HMM and thus made similar predictions about behavior, the precision signals generated by these two algorithms are quite distinct and can therefore be compared against neural activity in the amygdala. In a direct comparison, activity in the amygdala was best accounted for by the precision signal generated by the full HMM. It is interesting to note that evidence for model-based processing in the amygdala was more robust in the aversive case given the traditional view of the amygdala as being associated especially with aversive processing. However, it is unlikely that this pattern of results reflects a qualitative difference in the way that appetitive and aversive learning is mediated by the amygdala, particularly in the light of considerable evidence implicating this structure in both reward-related as well as aversive-learning [28], [29].

Finally, we checked the correlation between the precision signal we found here and an associability signal generated by the Pearce-Hall learning rule, and we found the correlation between these signals to be essentially negligible (with r ranging from −0.06 to −0.14), as opposed to being strongly negatively or positively correlated as would be anticipated were these signals to tap similar underlying processes.

The fact that in the present study we found model-based signals in the amygdala does indicate that this structure is capable of performing model-based inference even during Pavlovian conditioning. However, it is important to note that the findings of the present study do not rule out a role for this structure in prediction error driven model-free computations during Pavlovian conditioning. Indeed, while the prediction error driven model-free learning rules we used did not work very well in accounting for behavior on the task (as indexed by changes in reaction times), we did find some evidence (albeit weakly) of model-free value signals in the amygdala as generated by either a Rescorla-Wagner, a Pearce-Hall or a Hybrid learning rule. Indeed, while using our HMM model we did not find evidence for aversive-going expected value signals in the aversive session (i.e. by showing an increase in activity the more the unpleasant tasting liquid was expected), we did find such a signal correlating with expected value as computed by a Pearce-Hall learning rule. As a consequence, we cannot rule out a contribution for the amygdala in model-free computations. It is important to note however, that in many tasks in which neuronal activity was found in the amygdala to correlate with the predictions of model-free learning algorithms [18], [30]–[32], such tasks were either not set up to discriminate the predictions of model-free versus model-based learning rules, or else the relevant model comparisons were not performed. Thus, it is entirely feasible that many of the computations found in the amygdala in previous studies correspond more closely to model-based as opposed to model-free learning signals. More generally, if indeed, both model-based and model-free signals are present in the amygdala during Pavlovian conditioning, then an important question for future research will be to address how and when these signals interact with each other.

To conclude, we have found in the present study evidence for the existence of model-based learning signals in the human amygdala during performance of a Pavlovian conditioning task with a simple task structure. These findings provide an important new perspective into the functions of the amygdala by suggesting that this structure may participate in model-based computations in which abstract knowledge of the structure of the world is taken into account when computing signals leading to the elicitation of Pavlovian conditioned responses. The findings also resonate with an emerging theme in the neurobiology of reinforcement learning whereby value signals are suggested to be computed via two mechanisms: a model-based and a model-free approach [1], [3]. Whereas up to now, theoretical and experimental work on this distinction has tended to be focused on the domain of instrumental conditioning [4], [7], [8], the present study illustrates how similar principles may well apply even at the level of Pavlovian conditioning. Thus the distinction between model-based and model-free learning systems may apply at a much more general level across multiple types of associative learning in the brain. Furthermore, the present results provide evidence that model-based computations may be present not only in prefrontal cortex and striatum, but also in other brain structures such as the amygdala.

## Materials and Methods

### Subjects

Nineteen right-handed subjects (8 females) with a mean age of 22.21±3.47 participated in the study. All subjects were free of neurological or psychiatric disorders and had normal or correct-to-normal vision. Written informed consent was obtained from all subjects, and the study was approved by the Trinity College School of Psychology research ethics committee.

### Task description

Subjects participated in a Pavlovian task where they had to learn associations between different cues (fractal images) and a pleasant (blackcurrant juice [Ribena, Glaxo-Smithkline, UK]), affectively neutral (artificial saliva made of 25 mM KCl and 2.5 mM NaHCO3) or unpleasant (salty tea made of 2 black tea bags and 29 g of salt per liter) flavor liquid. The task consisted of two sessions lasting approximately 22 minutes each. Each session was composed of 120 trials, leading to a total of 240 trials. In one of the sessions, subjects underwent an appetitive Pavlovian conditioning procedure whereby they were presented with cues leading to the subsequent delivery of either the pleasant flavor, or the affectively neutral one, while in the other aversive conditioning session, subjects underwent an aversive conditioning procedure whereby they were presented with cues leading to the subsequent delivery of either the unpleasant flavor stimulus, or else the affectively neutral stimulus. The rationale for including the appetitive and aversive conditioning procedures in separate sessions as opposed to including both conditions intermixed within the same sessions was to avoid contrast effects observed in prior behavioral piloting whereby cues signaling the aversive outcome tended to overwhelm cues signaling the pleasant one such that both the pleasant and the neutral cue stimuli were viewed as relief stimuli (contrasted against the aversive outcome) [33]. Performing the appetitive and aversive conditioning procedures in separate sessions ensured robust behavioral conditioning in both the appetitive and aversive cases and largely avoided contrast effects between the appetitive and aversive conditions.

For both sessions, on each trial, a cue was displayed randomly on either the left or right side of a fixation cross for 4 seconds. Following a well-established Pavlovian conditioning protocol [34]–[36], subjects were also instructed to indicate on which side of the screen the cue was presented by means of pressing the laterally corresponding button on a response box, yet they were also instructed that the subsequent outcomes were not contingent on their responses. This serves two purposes: it allows one to monitor the extent to which participants are paying attention to the cues on each trial, as well as offering a response time measure which can serve as an index of conditioning. The offset of the cue (after 4 seconds) was followed by delivery of one of the liquid flavor stimuli with a probability of 0.6, or else no liquid stimulus was delivered. The next trial was triggered following a variable 2–11 secs inter-trial interval.

At the beginning of each session, subjects were presented with two novel fractal cues (not seen before in the course of the experiment): which we will denote as cue 1 and cue 2. In the appetitive session, cue 1 predicted the subsequent presentation of the pleasant liquid 60% of the time (or no liquid delivery 40% of the time), while in the aversive session cue 1 predicted the delivery of the aversive liquid 60% of the time (or no liquid delivery 40% of the time). Cue 1 and cue 2 trials were presented in a randomly intermixed order. After 16 trials (8 trials of each type), a reversal of the cue-outcome associations was set to occur with a probability of 0.25 on each subsequent trial. The probabilistic triggering of the reversal after the 16th trial ensured that the onset of the reversal was not fully predictable by subjects. Once a reversal was triggered, cue 1 no longer predicted the appetitive or aversive outcome but instead was associated with delivery of the neutral outcome, while cue 2 now predicted the appetitive or aversive outcome. After another 16 trials (8 trials of each type) following the onset of the reversal, another event was triggered to occur with probability 0.25 on one of the subsequent trials: this time instead of a reversal, a completely novel pair of stimuli was introduced. One of these, cue 3, was now paired with the appetitive or aversive outcome, while cue 4 was now paired with the neutral outcome. These new cues were presented for a further 16 trials, and followed again after a probabilistic trigger of p = 0.25 on each subsequent trial with a reversal of the associations. After the reversal, a new set of cues were introduced according to the same probabilistic rule and this was followed again by a reversal. Thus in total, 3 unique pairs of stimuli were used in each session and each of these pairs underwent a single reversal (Figure 1a,b). A completely different set of cues were used for each session, so that subjects experienced a total of 6 pairs of fractal stimuli throughout the whole experiment.

Within each session, the presentation order of the affective and neutral cue presentations was randomized throughout, with the one constraint that the cue predicting the neutral tasting liquid delivery had to be delivered twice every four trials. This ensured that the appetitive and neutral cues, and aversive and neutral cues were approximately evenly distributed in their presentation throughout the appetitive and aversive sessions respectively. All fractal images were matched for luminance. The order of the sessions was counterbalanced across subjects so that half of the subjects started the experiment with the appetitive session and half of the subjects with the aversive session.

### Subject instructions

Before the conditioning session, subjects received the following task instructions:

“In each trial, an image will appear on the screen and may be followed by some liquid delivery. There are six different images per session. Each image will lead to either a pleasant, neutral or unpleasant tasting liquid. You will have to learn these associations. However, during the experiment, this may change (or reverse), making image 1 associated with the liquid of image 2 and image 2 associated with the liquid of image 1. This reversal may actually happen more than once during the experiment and you have to fully pay attention and realize that it has happened. These cues may change during the experiment, so that you will have to learn these associations again with these new cues (which may also reverse).

At the beginning of each trial, the image will either appear on the left or right side of the screen. You will have to press the left button of the response pad if the image appears on the left side, or the right button if it appears on the right side. It is important that you press the button because we need to record your response times, although the trial will carry on if you don't press any button.

At the beginning and end of each session, we will ask you to rate different images and liquids. You will also have to rate these images in the middle of each session.”

### Apparatus

The pleasant, neutral and unpleasant tasting liquids were delivered by means of three separate electronic syringe pumps positioned in the scanner control room. These pumps pushed 1 mL of liquid to the subject's mouth via ∼10 m long polyethylene plastic tubes, the other end of which were held between the subject's lips like a straw, while they lay supine in the scanner.

### Behavioral measures

#### Affective evaluations of the fractal images and liquids

Participants were asked to provide subjective ratings indicating their perceived subjective hedonic evaluation for each of the 6 pairs of fractal images that were displayed. This was done during the experiment before each session, in the middle of each session (during the scanning) and at the end of each session, by presenting a picture of the fractal alongside an instruction to rate the fractal for its pleasantness on a scale going from 1 (do not like at all) to 4 (strongly like). These ratings could therefore provide a behavioral measure of evaluative conditioning [24] at three different time points throughout the experiment. Furthermore, before and after the appetitive session, the pleasant and neutral liquids were rated for their subjective pleasantness using a scale ranging from −5 (very unpleasant) to +5 (very pleasant), and similarly the aversive and neutral liquids were rated before and after the aversive session.

#### Preference ranking test

Before the experiment started and after the experiment was over, participants were asked to make binary choices indicating their relative preferences for each of 16 different fractals (12 of which were included in the experiment; 6 each in the appetitive and aversive sessions respectively; while 4 of the fractals were not featured in either session). Each of the 16 fractals was paired with each other fractal. This test allowed us to estimate a preference ranking for each of the fractals, thereby potentially providing an additional even more direct behavioral metric of evaluative conditioning beyond the pleasantness ratings.

#### Pupillary dilation

Pupil diameter was continuously measured during scanning using an MRI compatible integrated goggle and infrared eye tracking system (NordicNeuroLab AS, Bergen, Norway). Pupil reflex amplitude has been shown to be modulated by arousal level and can therefore be used as a physiological index of conditioning [22]–[24]. Pupil measurements could not be taken from 9 participants because space constraints within the head-coil alongside variations in head size meant that in some individuals the eye-tracker could not fit them comfortably.

#### Fluctuations in respiration and heart rate

Estimates of heart rate and respiration were recorded using a pulse oximeter positioned on the forefinger of subjects' left hand and a pressure sensor placed on the umbilical region. The time courses derived from these measures were used as a further physiological index of conditioning as well as being used separately to remove physiological noise from the fMRI data analysis (see fMRI data analysis).

### Data acquisition

Functional imaging was performed on a 3T Philips scanner equipped with an 8-channel SENSE (sensitivity encoding) head coil. Since the focus of our study was on the amygdala, we only acquired partial T2*-weighted images centered to include the amygdala while subjects were performing the task. These images also encompassed the ventral part of the prefrontal cortex, the ventral striatum, the insula, the hippocampus, the ventral part of the occipital lobe and the upper part of the cerebellum (amongst other regions). Nineteen contiguous sequential ascending slices of echo-planar T2*-weighted images were acquired in each volume, with a slice thickness of 2.2 mm and a 0.3 mm gap between slices (in-plane resolution: 1.58×1.63 mm; repetition time (TR): 2000 ms; echo time (TE): 30 ms; field of view: 196×196×47.2 mm; matrix: 128×128). A whole-brain high-resolution T1-weighted structural scan (voxel size: 0.9×0.9×0.9 mm) and three whole-brain T2*-weighted images were also acquired for each subject. To address the problem of spatial EPI distortions which are particularly prominent in the medial temporal lobe (MTL) and especially in the amygdala, we also acquired gradient field maps. To provide a measure of swallowing motion, a motion-sensitive inductive coil was attached to the subjects' throat using a Velcro strap. The time course derived from this measure was used as a regressor of no interest in the fMRI data analysis. Finally, to account for the effects of physiological noise in the fMRI data, subjects' cardiac and respiratory signals were recorded with a pulse oximeter and a pressure sensor placed on the umbilical region and further removed from time-series images. We discarded the first 3 volumes before data processing and statistical analysis to compensate for the T1 saturation effects.

### Preprocessing

All EPI volumes (‘partial’ scans acquired while subjects were performing the task and the three whole-brain functional scans acquired prior to the experiment) were corrected for differences in slice acquisition and spatially realigned. The mean whole-brain EPI was co-registered with the T1-weighted structural image, and subsequently, all the partial volumes were co-registered with the registered mean whole-brain EPI image. Partial volumes were then unwarped using the gradient field maps. After the structural scan was normalized to a standard T1 template, the same transformation was applied to all the partial volumes with a resampled voxel size of 0.9×0.9×0.9 mm. In order to maximize the spatial resolution of our data, no spatial smoothing kernel was applied to the data. These preprocessing steps were performed using the statistical parametric mapping software SPM5 (Wellcome Department of Imaging Neuroscience, London, UK).

#### Amygdalae segmentation

Amygdalae Regions of Interest (ROIs) were manually segmented for each subject by a single observer using a pen tablet (Wacom Intuos3 Graphics Tablet) in FSL View (FSL 4.1.2). This program allows magnification and the simultaneous viewing of volumes in coronal, sagittal and horizontal orientations. Amygdalae were manually outlined on each coronal image containing the amygdala using detailed tracing guidelines based on the Atlas of the Human Brain [25]. Outlines were checked in horizontal and sagittal planes when they proved more valuable for the identification of structure boundaries. The anterior limit of the amygdala was defined using the horizontal and sagittal planes. The following guidelines were used: In its rostral part, the amygdala is bordered ventromedially by the entorhinal cortex, ventrally by the temporal horn of the lateral ventricle and subamygdaloid white matter and laterally by white matter of the temporal lobe. Midrostrocaudally, the amygdala increases in size and is bordered ventromedially by a thin tract of white matter separating the amygdala and the entorhinal cortex, laterally by the white matter of the temporal lobe and medially by the semiannular sulcus. Caudally, the amygdala is bordered dorsally by the substantia innominata and fibers of the anterior commissure, laterally by the putamen, ventrally by the temporal horn of the lateral ventricle and the alveus of the hippocampus and medially by the optic tract.

#### Amygdalae normalization

Because structures in the MTL exhibit significant inter-individual anatomic variability, the signal-to-noise ratio in group analyses is substantially limited in this area [37]. Atlas-based approaches used to register whole-brain EPI images across subjects (such as SPM) look for a global optimum alignment which is achieved under the limitations imposed by the available degrees of freedom, and which is at the expense of regional accuracy. Consequently, BOLD signals in the MTL may be underestimated or possibly missed [38]. Alignment of the MTL is substantially improved by a ROI-alignment (ROI-AL) approach, where segmentations of regions of interest (ROIs) are drawn on structural images and aligned directly, resulting in an increased statistical power [39]. The last iteration of this alignment tool is ROI-Demons, which has proven to be exceptionally accurate in the alignment of hippocampal subfields for instance (http://darwin.bio.uci.edu/~cestark/roial/roial.html). Thirion's original demons algorithm has been implemented by Vercauteren and enforces smooth deformations by operating on a diffeomorphic space of displacement fields [40], [41]. Here, we used the implementation of ROI-Demons in the DemonsRegistration command-line tool (http://www.insight-journal.org/browse/publication/154). Our segmented amygdalae ROIs were registered with our amygdalae template based on 20 subjects from a previous study [18] to serve as an initial model and to align all amygdalae using DemonsRegistration. The resulting registered amygdalae were then averaged in SPM5 (using ImCalc) to create a first model. Subsequently, the initial non-registered amygdalae were registered with this first model and the newly registered amygdalae were averaged to create a second model. We repeated the last two steps three more times in order to generate a more accurate model. We finally registered our initial amygdalae ROIs with the fifth model to generate the resulting displacement fields (or transformation calculations). These individual displacement fields were then applied to each subject's normalized EPI scans in order to specifically normalize their amygdalae to our template amygdalae. We applied the same transformation to each subject's structural scan before averaging all the aligned structural scans, to create an amygdalae-aligned average structural brain of our 19 subjects. Finally, amygdalar subdivisions were hand-drawn on our template amygdalae using the Atlas of the Human Brain [25]. We delineated three sub-areas within the amygdala: the basolateral complex comprised of the basomedial, basolateral and lateral nuclei; the centromedial complex comprised of the central and medial nuclei; and the cortical complex (or cortical nucleus). In its most rostral part, the amygdala is exclusively composed of the basolateral complex. The cortical nucleus appears in the dorso-medial part of mid-rostral amygdala. The centromedial complex appears slightly more caudally than the cortical nucleus in the most dorsal part of the amygdala. The basolateral complex increases in size as one moves caudally from the anterior amygdala, has its maximal size midrostrocaudally and then decreases as one moves further back toward the caudal amygdala, whereas the cortical nucleus and centromedial complex slightly enlarge midrostrocaudally, but do not decrease in size as one moves further caudally within the amygdala. The cortical nucleus ends midcaudally, the basolateral complex ends in caudal amygdala while the centromedial complex ends in the most caudal part of amygdala.

### Computational model analysis

To test whether amygdala activity was better explained by model-based or model-free learning algorithms, we correlated brain activity in this region with expected value signals estimated by a number of different computational models. In model-free learning algorithms, the agent is surprised when a reversal occurs and starts learning again after it happens, whereas in model-based learning algorithms, the agent expects the reversal and considers it as resolution of uncertainty and does not need to relearn. The two modes of learning are diametrically opposed in the current task, therefore allowing us to test whether amygdala is tracking model-based or model-free computations.

#### Model-based learning algorithm: HMM with dynamic expectation of change

For the model-based learning algorithm, we used a Hidden Markov Model (HMM). In this HMM, the inferred state of the environment is defined in terms of an association between cues and outcomes and is represented by the psychological variable *S*. There are three possible liquid outcomes in the experiment (pleasant and neutral in the appetitive session and unpleasant and neutral in the aversive session) and two cues on any given trial. The state values *S_t_* are the possible combinations of cues and outcomes, for example *S_t_ = (cue 2, neutral liquid)*. Although the subjects were unaware that pleasant and unpleasant outcomes could not be delivered concurrently, this possible state value was omitted since it did not affect the results of the analyses. We also incorporated a binary-valued variable *H* in this HMM. The values of this hidden node determine whether *(H = 1)* or not *(H = 0)* the subject is expecting a reversal. A third random variable *O* represents the observed cue-outcome combination (see Figure 1d for a simple graphical representation of the model).

The transition probabilities of the reversal variable *H* are:
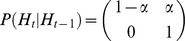
Variable values are enumerated along the row and column axes. Each entry of the matrix represents the probability of moving from one value on trial *t−1* (rows) to another on trial *t* (columns). At position (1,2), the α parameter is the probability of moving to the state of expecting a reversal *(H = 1)* from the *H = 0* state. Once a subject begins expecting a reversal, they do not switch back. This is encoded in the asymmetry of the transition matrix. The time evolution of *H* represents a subject's growing expectation of a reversal in the cue-outcome association. After the presentation of a novel pair of cues, *H* is set to the zero state. The transitions for the state variable *S* are conditionally dependent on the reversal variable:
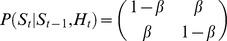
State reversals are inferred with a non-zero probability *β* when *H* is in the reversal expectation state *(H_t_ = 1)*, otherwise *β = 0* and *P(S_t_|S_t-1_,H_t_ = 0)* is the identity matrix. Note that after the first trial following the presentation of novel cues, the subject has a nonzero probability of being in the reversal expectation state thus they are always expecting a reversal to some degree and are prepared to react to an observation indicative of a contingency reversal. The posterior probability distribution *P(S_t_)* over the state values on trial *t* is determined by the prior state probability distribution *P(S_t-1_)*, the cue-outcome observation *O_t_*, and the state transition probabilities: 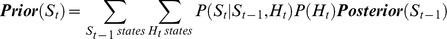


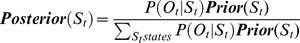
The prior over the state values at the beginning of a new set of cues is uniform. Beliefs are updated based on the likelihood of observing an outcome for a given cue and assuming a state such as “cue j is rewarding and this is likely to reverse soon.” For instance, if no reward is observed for cue j, then this state is given less credence because the likelihood that this occurs is low (0.4), and the expectation of reward for cue j is decreased. Significantly, expectations for the other cue are updated simultaneously, even if it is not implicated in the current trial. This is because a lower chance for the state “cue j is rewarding and this is likely to reverse soon” implies that the state “the other cue is rewarding and this is unlikely to reverse soon,” is more likely, and hence, the mathematical expectation of the reward upon presentation of the other cue increases.

The expected reward *Q_j_* when presented with a given cue j is
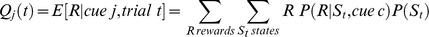
The reward *R* takes the values −1, 0, 1 for unpleasant, neutral, and pleasant rewards respectively. Here, “E” denotes the mathematical expectation operator. This means that the forecast is correct on average for all possible outcomes given a specific history of past rewards for both cues.

Confidence in, or precision about, the identity of the current state can be measured by the extent to which there are differences in the posterior probabilities of the possible states given past experience and the cues presented. When these differences are high, one posterior probability is necessarily high, and hence, precision is high. Conversely, if all posterior probabilities are the same, precision is lowest. We measure precision on a given trial *t* using the inverse Shannon entropy of the posterior distribution of the state variable *S*

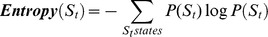
As more and more trials with no reward are experienced, the *H* node inputs a growing uncertainty about the identity of the current state into the HMM (since a reversal may have occurred in the absence of a rewarding outcome). Every time a new pair of cues is presented, precision is low but increases dramatically when the agent knows what particular state they are in (i.e. what the cue-liquid association is). Precision lowers again until the agent knows that a reversal has occurred, after which precision increases again. A random effects Bayesian approach was used for parameter fitting and model comparisons (note that we excluded one subject who failed to make motor responses from this analysis). Model parameters (such as α and β) were fixed a priori and the model fits were not sensitive to the specific values of these parameters. HMM estimation was performed via forward smoothing using the HMM toolbox for MATLAB (http://www.cs.ubc.ca/~murphyk/Software/HMM/hmm.html).

#### Model-free learning algorithms

*A. Rescorla Wagner algorithm.* In the Rescorla Wagner (RW) model, the new expected value at trial t+1 for a given cue is based on the sum of the current expected value and the prediction error between the reward obtained and the expected value at time t, weighted by the learning rate [11]:
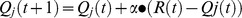
When j is a given cue, α is the learning rate with a range 0≤α≤1, and R(t) is the reward received on the current trial. If the valenced (pleasant or unpleasant) liquid was obtained on the current trial, R(t) = 1, else R(t) = 0. Hence there is one free parameter in this model, α. Note that using a random effects approach, we found that the optimal free parameters in the appetitive and aversive sessions averaged across subjects were 0.54 (SEM = 0.09) and 0.18 (SEM = 0.05) respectively.

*B. Pearce Hall algorithm.* This model differs from the Rescorla Wagner model (RW) in that it introduces an associability component and allows the effectiveness of the reinforcer to remain constant throughout conditioning. The associability values estimated by this model will decrease as the consequences of the conditioned stimulus become accurately predicted [12]. The expected values Q(t) of a given cue were updated according to:
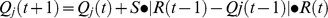
When j is a given cue, S is a free parameter governing the intensity of the CS, and R(t) is the reward received on the current trial. If the valenced (pleasant or unpleasant) liquid was obtained on the current trial, R(t) = 1, else R(t) = 0. In the Pearce Hall model (PH), the new expected value at trial t+1 for a given cue is based on the sum of the current expected value and the product of the absolute value of the difference between the outcome obtained on the previous trial and the expected reward on the previous trial, and the outcome obtained on the current trial; this product is weighted by the free parameter. Hence there is one free parameter in this model, *S*. Note that using a random effects approach, we found that the optimal free parameters in the appetitive and aversive sessions averaged across subjects were 0.58 (SEM = 0.09) and 0.40 (SEM = 0.10) respectively.

*C. Hybrid algorithm.* In addition to the Rescorla-Wagner and Pearce-Hall models, we also tested a hybrid model introduced by Li et al., (2011) in which the Rescorla-Wagner rule is used to update value expectations, while the Pearce-Hall rule is used to set the learning rate. The expected values Q(t) of a given cue were updated according to:
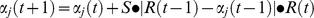



*D. Reduced HMM.* In order to set an even stronger test for our model-based HMM, we constructed a simpler version of the HMM. In this version of the HMM, *H* is always set to the *H = 1* state and thus the chance of a reversal happening is constant over time. As a result, in this HMM, there is no change in the expectation of when a reversal is going to occur over the course of a trial. In that sense this reduced model behaves more like the model-free algorithms, although it still incorporates knowledge of the CS-US state-space structure (namely, that one CS is paired with an affectively significant outcome while the other is associated with a neutral outcome), the algorithm cannot be said to be completely model-free in the same way as the prediction-error driven learning rules described above.

The expected reward signals from the reduced HMM are very similar to that generated by the full model-based HMM (with correlations of r = 0.987 in the appetitive session, r = 0.986 in the aversive session). Nevertheless, the precision signals generated by the reduced HMM are very different to those generated by the model-based HMM. Precision starts low every time a new pair of cues is presented and increases substantially when the agent knows in which state they are, but because the chance of a reversal occurring does not increase over time, the precision remains high through the rest of the learning with that cue until a new pair of cues is introduced. In other words, there is no decrease in precision related to the anticipation of a change in the contingencies (which would come from having a model of when the contingencies are predicted to reverse), but instead a decrease in precision occurs only once a contingency change has occurred and been detected through trial and error experience.

*E. Baseline model.* Our baseline model simply assumes that rewards occur completely at random and no learning takes place. Hence, expected values for all trials are kept at a constant value of 0.5.

### Model comparison on behavioral data

To perform a formal model comparison on the behavioral conditioning data, we used the trial-by-trial reaction time data (measuring the length of time taken on each trial for participants to press a button to indicate which side of the screen the Pavlovian cue stimulus had been presented). Many previous studies have shown that changes in RTs to a Pavlovian cue are correlated with changes in associative encoding between cues and behaviorally significant outcomes [13], [34], [36], [42]. For each session separately, we log transformed and adjusted the RT data to account for a linear trend in RTs over time independently of trial type, as well as to remove the effects of changes in reaction time related to switching responses from one side of the screen to the other. This was done by regressing the log transformed RTs against a matrix containing a column of ones, a column accounting for the linear trend over time and a column indicating whether participants switched their response from left to right or vice versa between the current and previous trial using the function regress in Matlab.

Using the same function, we then regressed these adjusted response times against the expected values generated by our model-based HMM our model-free RW, PH and Hybrid algorithms and our baseline model (for the baseline model, a small amount of noise was added to each expected value in order to compute the regression; without any noise the regression would not be calculable). This second regression analysis was run for each of these models, and cycled through all the possible learning rate parameters for the RW model and CS intensity parameters for the Pearce-Hall and hybrid models between 0 and 1, with increments of 0.001. This method returned Sum Squared Error (SSE) values for each of these parameter values thereby allowing us to obtain the best fitting value for the free parameter for the appetitive and aversive sessions (i.e. the free parameter associated with the lowest SSE value). In order to compare the model goodness between these four different algorithms, we converted the best SSE value of each session (appetitive and aversive) and each model into a Bayesian information criterion (BIC) value. The BIC adds a penalty proportional to the number of additional free parameters to the SSE value of each model, depending also on the number of degrees of freedom which in this case, is the total number of trials per session across all subjects [43]. Using this procedure, we found that in both the appetitive and aversive sessions, the model-based HMM outperformed the prediction-error driven model-free algorithms (Table 1). In the model validation analyses, where we compared the prediction-error driven models against a random baseline model, only the model-based HMM fit our behavioral data significantly better than the baseline model (Table 2). Hence, unlike RW, PH and the Hybrid model, the model-based HMM predicted RTs better than chance performance. Note that we did not regress the expected values generated by our reduced HMM since they were highly correlated with that of our model-based HMM.

### fMRI data analysis

The event-related fMRI data were analyzed by constructing sets of δ (stick) functions at the time of cue presentation and at the time of outcome for the appetitive and aversive sessions. For our main GLM (illustrated in Figures 4 and 5), additional regressors were constructed by using the expected values and the precision values generated by the model-based HMM as modulating parameters at the time of cue presentation. In order to compare model-based versus model-free learning algorithms in the amygdala, we ran three additional GLMs. For RW, the regressors were similar to our model-based HMM except that we did not have a regressor for precision which is not estimated by RW, and we added a modulating parameter for prediction error at the time of outcome. The regressors used in the GLM computed using PH model were the same as the ones used in our model-based HMM, except that the precision modulating parameter was replaced with an associability modulating parameter at the time of cue presentation (note that similar regressors were used for the Hybrid model). Finally, we ran an analysis using our reduced model HMM using the same regressors as for our model-based HMM. All of these regressors were convolved with a canonical hemodynamic response function (HRF). The six scan-to-scan motion parameters derived from the affine part of the realignment procedure were included as regressors of no interest to account for residual motion effects. To account for motion of the subjects' throat during swallowing, we added a regressor of no interest for swallowing motion. Finally, we also included thirteen additional regressors to account for physiological fluctuations (4 related to heart rate, 9 related to respiration) which were estimated using the RETROICOR algorithm [44]. Six of the 38 (2 sessions×19 subjects) log files could not be used to estimate these regressors due to a technical problem during data collection, and the missing physiological regressors were simply omitted for those sessions. All of these regressors were entered into a general linear model and fitted to each subject individually using SPM5. The resulting parameter estimates for regressors of interest were then entered into second-level one sample t-tests to generate the random-effects level statistics used to obtain the results shown in figures 4 and 5. All reported fMRI statistics and p values arise from group random-effects analyses. We present our statistical maps at a threshold of p<0.005, corrected for multiple comparisons at p<0.05. To correct for multiple comparisons, we first used the 3dFWHMx function in AFNI to estimate the intrinsic smoothness of our data, within the area defined by a mask corresponding to our amygdala template. We then used the AlphaSim function in AFNI to estimate via Monte Carlo simulation an extent threshold for statistical significance that was corrected for multiple comparisons at p<0.05 for a height threshold of p<0.005 within the amygdala ROI.

### Model comparison on BOLD data

In order to test whether amygdala activity was better accounted for by the model-based than model-free learning algorithms, we used a Bayesian model selection procedure (BMS) [45]. For both the appetitive and aversive sessions, we included in this model comparison individual betas averaged across voxels within a 4 mm sphere centered on the peak voxels of the amygdalar activities correlating with either expected value signals for the HMM or the model-free algorithms using the leave-one out method, thereby avoiding a non-independence bias in the voxel selection [46]. Using the spm_BMS function in SPM8, we compared expected value signals across all model-based (HMM) and model-free models separately for the appetitive and aversive sessions.

We used a similar approach to compare neural activity pertaining to precision signals estimated by our model-based and reduced model HMMs. The difference between these two HMMs is that the model-based HMM does not allow for a reversal without moving from a “non-reversal state” to a “possible reversal state”. As a consequence, the precision values generated by these models are clearly distinguishable and thus easily comparable using a BMS (whereas the estimated expected rewards are strongly correlated). Again, we included in this model comparison voxels within a 4 mm sphere centered on the peak voxels of the amygdalar activities correlating with precision signals for either the model-based HMM or the reduced model HMM using the leave-one out method. Here, we compared activity correlating with precision signals between the model-based and reduced HMM separately for the appetitive and aversive sessions (see Results section for the exceedance probabilities).

### ROI analyses

Functional regions of interest (ROIs) were defined using the MarsBaR toolbox (http://marsbar.sourceforge.net/). Beta estimates were extracted for each subject from the functional clusters of interest as they appeared on the statistical maps of a given contrast using the leave-one out method to avoid a non-independence bias. They were then averaged across subjects to plot expected reward (Figure 4b) and precision (Figure 5b) according to 3 categories (category 1 corresponding to the lowest values and category 3 corresponding to the highest values).
